# Examining the source of increased bipolar disorder and major depressive disorder common risk variation burden in multiplex schizophrenia families

**DOI:** 10.1038/s41537-022-00317-w

**Published:** 2022-11-25

**Authors:** Mohammad Ahangari, Robert Kirkpatrick, Tan-Hoang Nguyen, Nathan Gillespie, Kenneth S. Kendler, Silviu-Alin Bacanu, Bradley T. Webb, Brian C. Verrelli, Brien P. Riley

**Affiliations:** 1 Virginia Institute for Psychiatric and Behavioral Genetics, Virginia Commonwealth University, Richmond, VA USA; 2 Integrative Life Sciences PhD Program, Virginia Commonwealth University, Richmond, VA USA; 3 Department of Psychiatry, Virginia Commonwealth University, Richmond, VA USA; 4 Department of Human and Molecular Genetics, Virginia Commonwealth University, Richmond, VA USA; 5 GenOmics, Bioinformatics, and Translational Research Center, Biostatistics and Epidemiology Division, RTI International, Research Triangle Park, NC USA; 6 Center for Biological Data Science, Virginia Commonwealth University, Richmond, VA USA

**Keywords:** Schizophrenia, Psychosis

## Abstract

Psychotic and affective disorders often aggregate in the relatives of probands with schizophrenia, and genetic studies show substantial genetic correlation among schizophrenia, bipolar disorder, and major depressive disorder. In this study, we examined the polygenic risk burden of bipolar disorder and major depressive disorder in 257 multiplex schizophrenia families (*N* = 1005) from the Irish Study of High-Density Multiplex Schizophrenia Families versus 2205 ancestry-matched controls. Our results indicate that members of multiplex schizophrenia families have an increased polygenic risk for bipolar disorder and major depressive disorder compared to population controls. However, this observation is largely attributable to the part of the genetic risk that bipolar disorder or major depressive disorder share with schizophrenia due to genetic correlation, rather than the affective portion of the genetic risk unique to them. These findings suggest that a complete interpretation of cross-disorder polygenic risks in multiplex families requires an assessment of the relative contribution of shared versus unique genetic factors to account for genetic correlations across psychiatric disorders.

## Introduction

Psychotic and affective disorders have long been viewed as two separate axes of mental illness, and early practitioners of psychiatry like Emil Kraepelin and Eugen Bleuler observed that relatives of patients with schizophrenia (SCZ) have an increased rate of psychiatric disorders ranging from atypical psychoses to SCZ spectrum personality disorders, many of which appeared to be milder versions of the symptoms observed in patients with SCZ^1^. Some of the first family studies of SCZ conducted in the early 20th century, confirmed that in addition to SCZ, a range of other psychiatric disorders on the psychosis spectrum also aggregate in the relatives of probands with SCZ. These findings were later solidified by the Danish Adoption Study of Schizophrenia, which showed that biological relatives of patients with SCZ were at an increased risk for SCZ as well as milder syndromes on the psychosis spectrum^2^.

Large-scale genome-wide association studies (GWAS) conducted by the Psychiatric Genomics Consortium (PGC) have shown that common risk variation in the genome (minor allele frequency ≥1%) can explain a modest portion of the heritability of major psychiatric disorders^3–5^. Additionally, the Cross-Disorder Group of the PGC has provided robust, replicable evidence for strong genetic correlation (*r*_G_) among SCZ and bipolar disorder (BIP) (*r*_G_ = 0.68), and SCZ and major depressive disorder (MDD) (*r*_G_ = 0.35)^6^. BIP and MDD also have a significant positive genetic correlation estimated to be *r*_G_ = 0.44^4^. Together, these results indicate that there is a substantial genetic overlap among these three disorders with varying degree of psychotic and affective features, suggesting widespread pleiotropy in the genetic architecture of psychiatric disorders at common variation level^6^.

The Irish Study of High-Density Schizophrenia Families (ISHDSF)^7^ consists of 257 multiplex SCZ families with genotype data, ascertained to have two or more first-degree relatives meeting the Diagnostic and Statistical Manual of Mental Disorders (DSM-III-R) criteria for SCZ or poor-outcome schizoaffective disorder. In line with previous epidemiological observations in the relatives of probands with SCZ^8,9^, in addition to a significant aggregation of psychotic disorders, other psychiatric diagnoses including affective, personality, and substance use disorders, are also present in the ISHDSF sample^7^. Furthermore, our previous polygenic risk score (PRS) profiling in the ISHDSF sample shows that all case definitions of the psychosis spectrum in the ISHDSF sample, including the unaffected relatives of probands, have an increased burden of common SCZ risk variation compared to population controls. This observation is consistent with its polygenic architecture, and the observation of SCZ transmission through some non-psychotic, or unaffected family members in this sample^10,11^.

The high baseline risk for SCZ observed across all case definitions of the ISHDSF sample^10^, coupled with the evidence for strong *r*_G_ among SCZ, BIP, and MDD, suggests that members of the ISHDSF may have an increased polygenic risk for BIP and MDD. In this study, we sought to test this hypothesis by constructing univariate BIP and MDD PRS in subjects from 257 multiplex SCZ families and 2,205 ancestry-matched population controls all from the population of the island of Ireland. Given that the strong *r*_G_ among SCZ, BIP, and MDD, makes standard univariate cross-disorder PRS profiling in members of multiplex families less informative, we also used GWAS-by subtraction^12^ as implemented in the genomic structural equation modeling (genomicSEM) framework^13^, to disentangle BIP and MDD polygenic signals into underlying genetic factors. By doing so, we investigated whether the increased polygenic risk for BIP or MDD in multiplex SCZ families is attributable to the portion of the genetic risk that these two disorders share with SCZ due to their *r*_G_, or the affective portion of the genetic risk that is unique to them. To further investigate whether polygenic risks for BIP and MDD, and their unique and shared genetic factors are over-transmitted from parents to probands in the families, we performed polygenic transmission disequilibrium testing^14^ in a subset of the ISHDSF sample with full parent-offspring information. Based on epidemiological findings in multiplex SCZ families and the substantial r_G_ among SCZ, BIP, and MDD, we hypothesized that increased burden of common risk variation conferring risk to BIP or MDD in multiplex SCZ families is likely to be due to the portion of the genetic risk that these disorders share with SCZ due to *r*_G_, rather than the affective portion of the genetic risk unique to them. Therefore, by addressing these questions, we attempt to clarify the complexity of cross-disorder PRS analyses in multiplex families.

## Results

### Sample structure

**Fig. 1:**
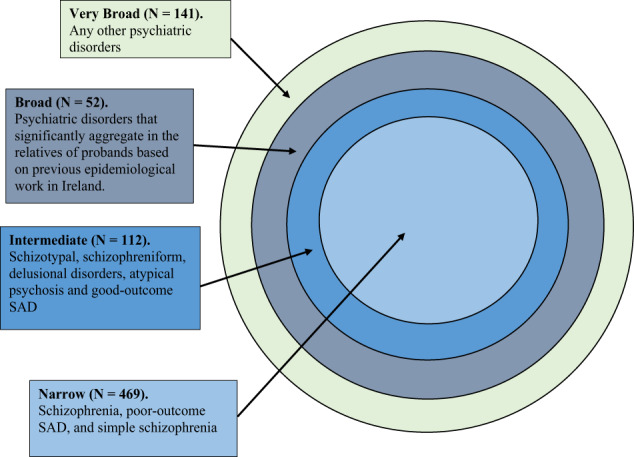
Concentric diagnostic schema of the ISHDSF sample. The concentric diagnostic schema of the ISHDSF sample based on previous genetic epidemiology contains four case definitions reflecting the schizophrenia spectrum: (1) *narrow* case definition, (2) *intermediate* case definition, (3) *broad* case definition, and (4) *very broad* case definition. SAD = schizoaffective disorder.

The diagnostic schema in the ISHDSF (Fig. 1) follows a concentric pattern ranked by the degree to which they reflect the narrow versus broad definitions of the psychosis spectrum. This includes four case definitions: *narrow* which includes SCZ, simple SCZ, and poor-outcome schizoaffective disorder, *intermediate* (adding schizotypal personality disorder, schizophreniform disorder, delusional disorders, psychosis not otherwise specified, and good-outcome schizoaffective disorder), *broad* (adding psychotic affective illness, paranoid, avoidant, and schizoid personality disorders, and other disorders that significantly aggregate in relatives of SCZ probands based on previous epidemiological work in Ireland^15^), and *very broad* (adding any other psychiatric illnesses in the families). The ISHDSF sample also includes *unaffected* relatives in multiplex families with no diagnosis of any psychiatric illness.

### Increased univariate BIP and MDD PRS in multiplex SCZ families

**Fig. 2:**
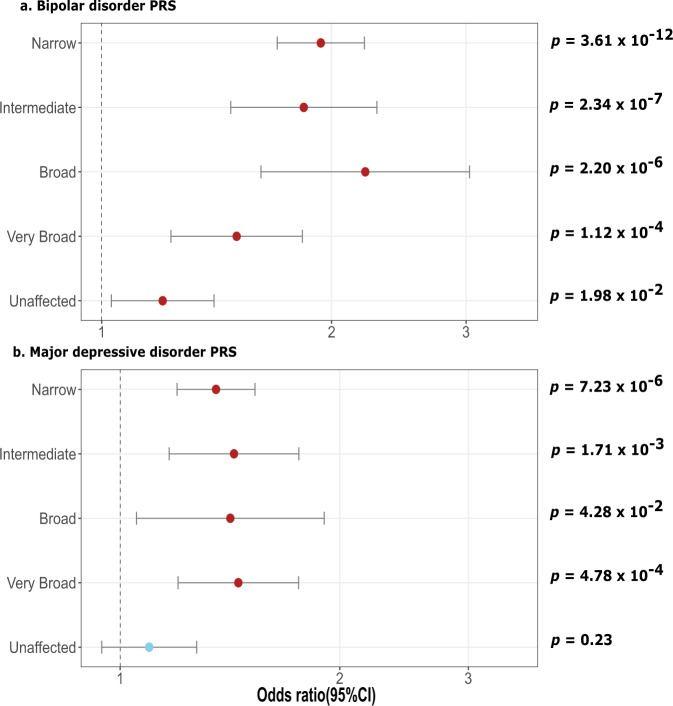
Odds ratio (OR) plots with 95% confidence intervals (95% CI) for bipolar disorder (BIP) and major depressive disorder (MDD) PRS. All comparison analyses follow the hypothesis that ISHDSF subjects have a higher PRS for BIP (**a**) or MDD (**b**) compared to population controls. All PRS have been normalized using *Z* score standardization prior to obtaining ORs. The plot shows OR (filled circles) with 95% CI for each case definition compared to population controls. One-sided *p* values after multiple testing corrections are provided on the right side of the *Y* axis. Red dot represents significant and the blue dot represents non-significant results.

Fig. 2 shows the results for the univariate BIP and MDD PRS in multiplex SCZ families versus population controls. All case definitions in multiplex SCZ families show a significantly increased BIP PRS compared to population controls (Fig. 2a). The highest odds ratio (OR) was observed in the *broad* case definition (OR = 2.21, 95% CI = 1.62–3.03) which includes 17 of the 21 BIP cases in the ISHDSF sample with psychotic features, excluding BIP cases without psychotic features which are represented in the *very broad* case definition. Except for the *unaffected* relatives, all case definitions in the ISHDSF sample also show a significantly increased MDD PRS compared to population controls (Fig. 2b). The highest OR was observed in the *very broad* case definition (OR = 1.45, 95% CI = 1.20–1.76), which includes 80 of 102 MDD cases in the ISHDSF sample, excluding MDD cases with psychotic features that are represented in the *broad* case definition. Full results are provided in Supplementary Tables 1 and 2.

### No increased affective factor PRS in multiplex SCZ families

**Fig. 3:**
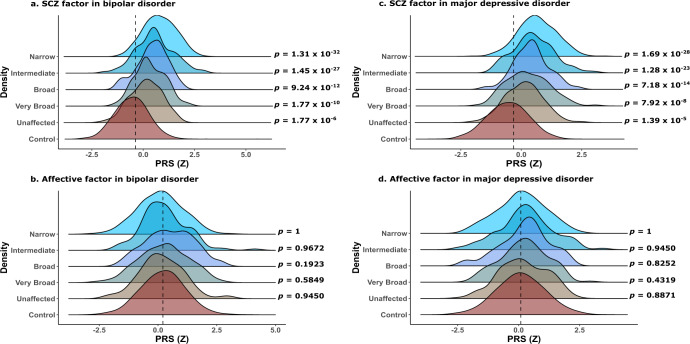
Density plots showing the distribution of SCZ factor and Affective factor PRS results. Dotted line shows the mean PRS value for population controls. **a**, **b** show the distribution of *SCZ* factor PRS for bipolar disorder (BIP) and major depressive disorder (MDD) respectively. **c**, **d** show the distribution of *Affective* factor PRS for BIP and MDD respectively. Each color represents one of the case definitions in the ISHDSF sample shown on the left side of each panel. One-sided *p* values after multiple testing correction are provided on the right side of each panel. All comparisons follow the hypothesis that ISHDSF members have higher PRS compared to population controls.

Fig. 3 shows the results for *SCZ* factor and *Affective* factor components derived from BIP and MDD polygenic risks in multiplex SCZ families versus population controls. The PRS constructed for *SCZ* factor in BIP, and *SCZ* factor in MDD (Fig. 3a, c), representing part of the polygenic risk that these two disorders share with SCZ due to *r*_G_, are significantly increased in all case definitions in the ISHDSF sample compared to population controls. In contrast, the PRS constructed from *Affective* factor in BIP, and *Affective* factor in MDD (Fig. 3b, d), representing the affective portion of the polygenic risk unique to these two disorders, show no significant increase in members of multiplex SCZ families compared to population controls. Full results are provided in Supplementary Table 3.

To assess the generalizability of these observations beyond multiplex SCZ families, we replicated the PRS analyses for *SCZ* and *Affective* factors underlying BIP and MDD in an independent cohort of ancestry-matched sporadic SCZ cases (*N* = 2225) from Ireland. As shown in Supplementary Fig. 1, the observed pattern of PRS enrichment in cases from multiplex SCZ families is similar to ancestry-matched sporadic cases, demonstrating the generalizability of these observations in an independent cohort of SCZ cases.

### Polygenic transmission disequilibrium test (pTDT) in multiplex SCZ families

**Fig. 4:**
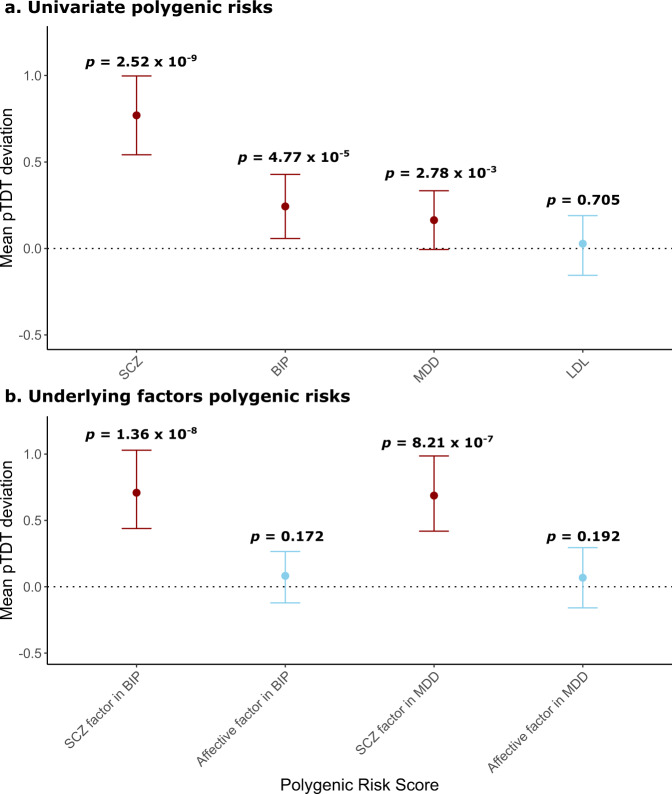
Polygenic transmission disequilibrium test results. transmission disequilibrium is presented as standard deviation on the mid-parent distribution with 95% confidence intervals. One-sided one-sample *t*-tests were used to evaluate polygenic over-transmission in 41 multiplex SCZ families. **a** polygenic risk for schizophrenia (SCZ), bipolar disorder (BIP), major depressive disorder (MDD), and low-density lipoprotein (LDL). **b** Polygenic risk for *SCZ* factors and Affective factors, representing part of the genetic risk shared with SCZ or unique to BIP or MDD, respectively. Dotted line represents mid-parent PRS.

Fig. 4 shows the pTDT results in multiplex SCZ families. In panel 4a we show that as expected, SCZ polygenic risks described in a previous study^10^ are significantly over-transmitted from parents to probands, while low-density lipoprotein (LDL) polygenic risks used as a negative control show no significant over-transmission from parents to probands, suggesting the absence of systematic biases in the results. We next show that univariate BIP and MDD polygenic risks are also significantly over-transmitted from parents to probands in multiplex SCZ families (Fig. 4a). Polygenic risks for *SCZ* factors derived from BIP and MDD are also significantly over-transmitted from parents to probands in the families. In contrast, no significant over-transmission of polygenic risks derived from *Affective* factors unique to BIP or MDD were observed in multiplex SCZ families (Fig. 4b).

### Polygenicity and polygenic overlap

**Fig. 5:**
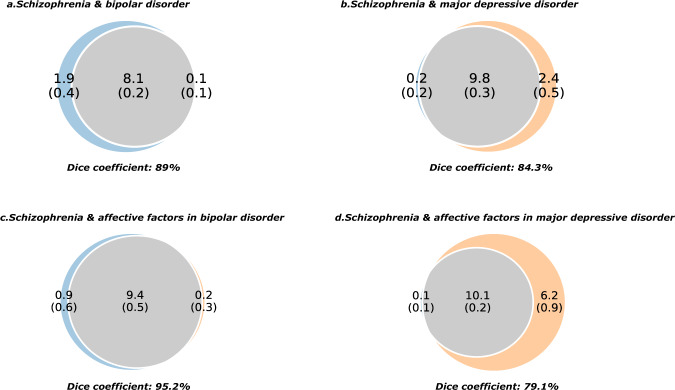
Polygenic overlap of schizophrenia (SCZ) with bipolar disorder (BIP) and major depressive disorder (MDD) and their derived latent factors. Venn diagrams show the estimated portion of non-null causal variants shared and unique to each of the phenotypes. The gray part represents the overlap, and the size represents the level of polygenicity for each phenotype. The numbers indicate the number of causal variants in thousands, explaining 90% of SNP heritability for each phenotype followed by the standard error in parenthesis. The polygenic overlap is measured as the Dice coefficient, which is the percentage of shared causal variants between two phenotypes (0–100% scale). **a** Polygenic overlap between schizophrenia and bipolar disorder. **b** Polygenic overlap between schizophrenia and major depressive disorder. **c** Polygenic overlap between underlying factors in bipolar disorder. **d** Polygenic overlap between underlying factors in major depressive disorder.

We used MiXeR^16^ to estimate polygenicity, polygenic overlap, and the number of shared and unique causal variants between SCZ and BIP, SCZ and MDD, and their underlying latent genetic factors. In agreement with previous findings, the polygenic signals of SCZ substantially overlap with BIP (Fig. 5a) and MDD (Fig. 5b), while SCZ is estimated to be more polygenic than BIP (Fig. 5a), but less polygenic than MDD (Fig. 5b)^16^. Similarly, we observe that polygenic signals from *SCZ* factors and *Affective* factors derived from BIP (Fig. 5c) and MDD (Fig. 5d) also substantially overlap. The *SCZ* factor underlying BIP (Fig. 5c) is estimated to be more polygenic than the *Affective* factor underlying BIP, whereas the *Affective* factor underlying MDD (Fig. 5d) is estimated to be more polygenic than the *SCZ* factor.

## Discussion

Large-scale GWAS of SCZ, BIP, and MDD^4,5,17^, have shown that many common risk variants with small effect sizes contribute to disease risk in major psychiatric disorders. Additionally, cross-disorder analyses of psychiatric disorders have also provided consistent evidence that SCZ, BIP, and MDD share substantial genetic risk at common variation level^6^. Based on these observations, we sought to investigate the source of the increased common risk variation burden of BIP and MDD, two disorders with prominent affective features and strong *r*_G_ with SCZ, in members of multiplex SCZ families.

Our results indicate that members of multiplex SCZ families, including the unaffected relatives, have an increased burden of common risk variation conferring risk to BIP compared to ancestry-matched population controls. With the exception of the unaffected relatives, we also observe that members of multiplex SCZ families also have an increased burden of common risk variation conferring risk to MDD compared to ancestry-matched population controls. We used genomicSEM to disentangle BIP and MDD polygenic risks into underlying genetic factors that they share with SCZ due to *r*_G_ which we called *SCZ* factors, and underlying genetic factors unique to BIP and MDD, which we called *Affective* factors. Our results suggest that increased polygenic risks for BIP and MDD in multiplex SCZ families are largely driven by part of the genetic liability that these two disorders share with SCZ due to *r*_G_. This observation is in agreement with epidemiological findings that show a significant increase in the incidence of psychotic, but not affective disorders in relatives of patients with SCZ in multiplex families^8,9,18^. While in addition to SCZ, non-schizophrenic psychotic disorders^19^ also show significant familial aggregation in multiplex families^15^, affective and anxiety disorders are generally not considered to be on the same continuum as psychotic disorders^20^. Therefore, these results provide empirical genetic evidence in support of previous epidemiological findings in multiplex SCZ families by suggesting that although members of multiplex SCZ families have an increased polygenic risk burden for two disorders with prominent affective features, the source of this increased polygenic risk in a sample with a high incidence of SCZ is largely due to strong r_G_ of these two disorders with SCZ. We also replicated these results in an independent sample of sporadic SCZ cases and showed that this observation is also generalizable to sporadic SCZ cases beyond multiplex families.

Using the MiXeR, we quantified the polygenicity and polygenic overlap between the latent factors generated in this study. MiXeR has been used in recent years to quantify the polygenicity and the polygenic overlap between SCZ and other complex traits^21–23^. In agreement with previous findings^16^, we first showed that SCZ shows substantial polygenic overlap with BIP and MDD. Next, we showed that while *SCZ* and *Affective* factors underlying BIP or MDD have a substantial overlap, *SCZ* factor appears to be more polygenic than *Affective* factor in BIP, whereas *SCZ* factor appears to be less polygenic than *Affective* factor in MDD. This observation is expected, as previous findings show that while SCZ is more polygenic than BIP, MDD is considered to be more polygenic than both BIP and SCZ^24^. Therefore, these findings suggest that although both BIP and MDD have prominent affective features in their etiology, the polygenic signals conferring risk to affective features of MDD appear to be more polygenic than BIP.

We used an extension of standard TDT, called pTDT to investigate whether polygenic risks for BIP, MDD, and their shared and unique genetic factors are over-transmitted from parents to probands. Children are expected to inherit half of their parents’ risk alleles and transmission disequilibrium method^25^ tests whether a genetic variant or aggregate polygenic scores in pTDT is transmitted more than half the time from parents to probands which signifies over-transmission. pTDT is robust to population stratification and different sources of unmeasured biases such as socioeconomic status or environmental influences since matched, un-transmitted chromosomes in families are employed as controls. In agreement with expectation and prior studies^26^, polygenic liability to SCZ was over-transmitted from parents to probands in the ISHDSF sample, while we observed no over-transmission of polygenic liability to LDL which shows no direct correlation or causation to psychiatric disorders. Next, we demonstrated that univariate BIP and MDD polygenic risks are also significantly over-transmitted from parents to probands in the ISHDSF sample, indicating that proband’s polygenic risks for these disorders were on average higher than that of their parents. Furthermore, only the portion of the polygenic risk that BIP or MDD share with SCZ due to *r*_G_ were over-transmitted from parents to probands, and no over-transmission of the affective portion of the risk unique to them was observed. Members of multiplex SCZ families show a higher incidence of psychotic, but not affective disorders compared to the general population, making these observations consistent with SCZ transmission through some non-psychotic or unaffected family members in the ISHDSF sample^10^.

Other families and pedigree studies of psychiatric disorders have also demonstrated the presence of an increased cross-disorder polygenic risk for psychiatric disorders. Andlauer and colleagues^27^ analyzed multiplex BIP families (*N* = 395) consisting of 166 BIP and 78 MDD cases and showed that familial BIP cases and their unaffected relatives, had an increased PRS for BIP and SCZ compared to population controls. Szatkiewicz and colleagues^28^ used a densely affected psychiatric pedigree (*N* = 418) and showed an increased SCZ PRS in affected members compared to unaffected members and population controls. De Jong and colleagues^29^ also used a dense pedigree (*N* = 300) with BIP and MDD cases and showed nominally significant BIP and SCZ PRS in affected members compared to unaffected members and population controls.

In contrast to the findings, Halvorsen and colleagues^30^ analyzed a densely affected pedigree (*N* = 122) with Tourette syndrome, a neurodevelopmental disorder characterized by recurrent nonrhythmic tics that shows significant *r*_G_ with obsessive-compulsive disorder (OCD) and attention deficit hyperactivity disorder (ADHD)^31,32^. While a significantly increased PRS for Tourette syndrome were observed in cases compared to controls, no significantly increased cross-disorder PRS for OCD or ADHD were observed. This lack of cross-disorder PRS loading in families with Tourette syndrome could reflect the low power of OCD^33^ and ADHD^34^ GWAS. Alternatively, there may be important differences in the genetic architecture of neurodevelopmental disorders such as Tourette syndrome that differentiate their polygenic profile from major psychiatric disorders such as SCZ. Here, we show that similar to the studies noted above^27–29^, members of multiplex SCZ families analyzed here (*N* = 1005) also have an increased cross-disorder polygenic risk for correlated psychiatric disorders. Furthermore, our PRS profiling in the full sample of multiplex SCZ families, in combination with pTDT results, provides empirical genetic evidence that the source of increased cross-disorder polygenic risk for BIP and MDD in multiplex SCZ families is attributable to the portion of the genetic risk that BIP or MDD share with SCZ. Despite distinct manifestations of psychotic and affective illnesses, these two separate axes of mental illness share significant genetic portions and our results offer new insights into the nature of the elevated risk for affective disorders such as BIP and MDD in multiplex SCZ families.

The analyses presented in this study should be interpreted in the context of some limitations. Although GWAS-by-subtraction can be extended from bivariate to multivariate models, we opted to use two separate bivariate models. This is due to our specific hypothesis about BIP and MDD as two distinct disorders with varying degrees of affective and psychotic features in their symptomatology. Additionally, caution is warranted due to the sample size and power limitations of current GWAS in psychiatric disorders. For example, if we extend our bivariate models to a single multivariate model and subtract out both BIP and MDD signals from SCZ in a single model, we may be left with inadequate signals for the affective factors since a large portion of signals would be subtracted out due to strong *r*_G_ among these three disorders. As the sample size and power of GWAS for psychiatric disorders increases, future work could extend the bivariate models to multivariate models in order to empirically test for this.

Testing the predictive power of *SCZ* and *Affective* factors in independent SCZ, BIP, and MDD cohorts could provide further genetic evidence in support of the derived factors from genomicSEM models. While we did not have access to independent BIP and MDD cohorts, we were able to show that *SCZ* factors underlying BIP and MDD can significantly distinguish between SCZ cases and controls from the ISGC cohort. Future work could also test the predictive power of *SCZ* and *Affective* factors in independent BIP and MDD cohorts.

Furthermore, some case definitions in the ISHDSF sample (e.g the *broad*), have a lower number of subjects, which may potentially bias some of the results due to lower power. We addressed this potential issue by repeating the PRS analyses by grouping the subjects in a concentric manner as described in the ISHDSF publication^7^. The concentric comparison versus population controls shows similar patterns of PRS enrichment, indicating that lower numbers of subjects in some of the diagnostic categories in the ISHDSF sample are unlikely to be a source of bias for the central findings (Supplementary Table 4). Given that environmental factors have not been assessed here, future analyses could also integrate environmental influences unique to the families to further elucidate the role of environmental factors on the elevated polygenic risk for BIP and MDD in multiplex SCZ families. The predictive power of current PRS methods is mostly limited to individuals of European ancestry. As cross-ancestry PRS methods become more sophisticated and samples of multiplex families from more diverse populations become available, we could replicate these findings in ancestrally diverse multiplex families. Additionally, PRS methods are limited to common risk variations in the genome which omits some important genetic risk factors in the genome. Despite sparse evidence for the involvement of rare risk variation in the genetic architecture of MDD^35^ and BIP^36^ at current sample sizes, copy number variants in the genome often show strong pleiotropy among psychiatric disorders^37^ and could influence the risk that is not captured by PRS profiling. Finally, due to the sample collection and genotyping strategies, not all the multiplex families have full parent-offspring genotypic or phenotypic information available. Therefore, pTDT analysis results should be interpreted with the caveat that 41 families out of the full sample of 257 had full parent-offspring and genotype information available.

In conclusion, in this study, we showed that in addition to the increased burden of common risk variation conferring risk to SCZ^10^, members of multiplex SCZ families studied here also have an increased polygenic vulnerability to BIP and MDD. However, this observation is likely to be largely attributable to part of the genetic risk that BIP or MDD share with SCZ due to their *r*_G_, rather than the affective portion of the genetic risk unique to these two disorders. Therefore, these results suggest that a complete interpretation of elevated cross-disorder PRS across correlated psychiatric disorders in multiplex families requires consideration of the relative contribution of the shared and unique genetic factors to account for the known genetic correlations across psychiatric disorders.

## Methods

### Sample description

#### Irish Study of High-Density Schizophrenia Families

Fieldwork for the ISHDSF sample was carried out between 1987 and 1992, with probands ascertained from public psychiatric hospitals in the Republic of Ireland and Northern Ireland^7^. Selection criteria were two or more first-degree relatives meeting DSM-III-R criteria for SCZ or poor-outcome schizoaffective disorder, with all four grandparents being born in either the Republic of Ireland or the United Kingdom. Relatives of the probands suspected of a psychotic illness were interviewed by trained psychiatrists, and trained social workers interviewed other relatives of the probands. To avoid bias and detect possible mistakes in diagnosis, independent review of all diagnostic information was made blind to family assignments by two trained psychiatrists, with each psychiatrist making up to 3 best estimate DSM-III-R diagnoses, with high agreement (weighted k = 0.94 + - 0.05). Full consent from all the participants and approval from local ethics committees in the Republic of Ireland and Northern Ireland, as well as Virginia Commonwealth University Institutional Review Board were acquired and archived and can be provided upon request.

#### Irish Schizophrenia Genomics Consortium (ISGC)

The ISGC sample was assembled for a GWAS of SCZ in Ireland. Details of recruitment, screening, and quality control (QC) of controls are described elsewhere^38^. Cases were recruited through community mental health services and inpatient units in the Republic of Ireland and Northern Ireland using DSM-III-R or DSM-IV following protocols with local ethics approval. Cases were further screened to exclude substance-included psychotic disorders. Controls were blood donors from the Irish blood Transfusion Service assembled as part of the Irish Biobank in the Republic of Ireland. All individuals reported all four grandparents were born in either Ireland or the United Kingdom, with no reported history of psychotic illness, and full consent was received from all the participants. Due to the relatively low lifetime prevalence of SCZ in the general population (~1%), misclassification of controls should have a minimum impact on power^39^.

### Genotyping and imputation

Information on genotyping and imputation is provided elsewhere^10^. Briefly, genotyping was carried out on 3 different arrays: 830 ISHDSF subjects were genotyped on the Illumina 610-Quad Array by Illumina, 175 ISHDSF subjects were later genotyped on the Infinium psychArray V.1.13 Array (psychArray) at Mount Sinai, and 1730 population controls were genotyped using the Affymetrix V.6.0 Array either at the Broad Institute or by Affymetrix. An additional 475 population controls that either did not pass the QC or were not included in the Affymetrix Array study were later genotyped on the psychArray along with the additional ISHDSF subjects at Mount Sinai. The same QC protocols were applied to all three genotype datasets. In brief, exclusion criteria for samples were a call rate of <95%, more than one Mendelian error in the ISHDSF sample, and a difference between reported and genotypic sex. Exclusion criteria for SNPs were MAF < 1%, call rate <98%, and *p* < 0.0001 for deviation from Hardy-Weinberg expectation. The final ISHDSF sample includes 1,005 subjects from 257 pedigrees whose SNP data from the Illumina Array and the psychArray passed all the QC filters. The final population control sample includes 2205 subjects whose SNP data from the Affymetrix and the psychArray passed all the QC filters.

Genotypes passing QC were phased using Eagle V.2.4 and imputed to the Haplotype Reference Consortium (HRC) reference panel using Minimac4 on the Michigan Imputation Server^40^. The HRC panel includes 64,975 samples that are predominantly of European ancestry, making it suitable for imputation of our sample from the island of Ireland^41^. Imputed genotype dosages were extracted and used for all downstream analyses. As part of the post-imputation QC, variants with MAF < 1% were excluded from the initial merging. After all standard QC measurements, 9,298,012 SNPs on the Illumina Array, 11,080,279 SNPs on the Affymetrix Array, and 11,081,999 SNPs on the psychArray remained for analysis. Of these three sets of SNPs, 9,008,825 SNPs were shared across all three imputed arrays and were used for downstream analyses. The imputation quality score for the shared SNPs used for PRS construction and downstream analyses was high across all three genotyping platforms (INFO ≥ 0.96 for all). More information on genotyping, imputation, and QC measures is provided in the Supplementary Materials, Supplementary Figs. 2–5, and Supplementary Tables 5 and 6.

### GWAS-by-subtraction

We performed GWAS-by-subtraction using the genomicSEM framework by analyzing summary statistics data for SCZ, BIP, and MDD. Leave-N-out SCZ GWAS excluding all Irish participants, (*N* = 156,509)^5^, BIP (*N* = 413,466)^4^, and MDD (*N* = 500,199)^17^ summary statistics were acquired and the genetic covariance between them was estimated using LD Score Regression^42^. SNPs were filtered for MAF < 0.01 and INFO < 0.8, and only SNPs that are present in both SCZ and BIP GWASs, or SCZ and MDD GWASs were used to generate the factors from GWAS-by-subtraction models. This left us with 6,361,243 SNPs for BIP, and 6,599,052 SNPs for MDD. We then used the post-QC summary statistics by first regressing them on two latent factors that we called *SCZ* factor and *Affective* factor underlying BIP or MDD. Next, we regressed *SCZ* factor and *Affective* factor on each SNP from the summary statistics that passed QC measurements as described above, which allowed for two separate paths of association with BIP or MDD for each SNP: (1) a path that is fully mediated by *SCZ* factor, and (2) a path that is fully independent of *SCZ* factor, called *Affective* factor. Therefore, *SCZ* factors in BIP or MDD capture part of the genetic risk that each of these two disorders shares with SCZ due to their *r*_G_, whereas *Affective* factors in BIP or MDD capture the affective portion of the genetic risk that is unique to BIP or MDD and not shared with SCZ. The models assume that genetic effects on SCZ are also impacting BIP or MDD to some degree given that both BIP and MDD have a strong genetic correlation with SCZ. The path diagrams for the Cholesky decomposition used to disentangle the polygenic signals are provided in Supplementary Fig. 6. More details on the GWAS-by-subtraction analysis, path estimates, and the formula used to estimate the effective sample size are provided in Supplementary Table 7 and Supplementary Materials.

### Construction of PRS

Summary statistics for BIP (*N* = 413,466)^4^, MDD (*N* = 500,199)^17^, *SCZ* factor in BIP (*N*_eff_ = 146,420), *Affective* factor in BIP (*N*_eff_ = 310,018), *SCZ* factor in MDD (*N*_eff_ = 147,014), and *Affective* factor in MDD (*N*_eff_ = 458,356) were used by excluding variants with MAF < 1% or imputation quality score of < 0.9 and removing strand ambiguous and indel polymorphisms. We then constructed PRS using a Bayesian regression framework by placing a continuous shrinkage prior to SNP effect sizes using PRS-CS^43^. Based on current recommendations^43^ we used the phi value of 1e-2 for BIP and MDD due to their high polygenicity, whereas the “auto” function was used to automatically learn the phi value for SNP weights for *SCZ* and *Affective* factors. PRS-CS uses linkage disequilibrium information from an external reference panel such as the 1000 Genomes European Phase 3 European sample^44^, to estimate the posterior effect sizes for each SNP. Although *p*-value thresholding and clumping method (P-T) have been traditionally used, PRS-CS shows substantial improvement in predictive power over P-T^45^.

### Polygenic transmission disequilibrium test

We used pTDT^14^ in a subset of the ISHDSF sample (41 families) with full parent-offspring data to test for over-transmission of polygenic risks for BIP, MDD, and their unique and shared (with SCZ) underlying genetic factors from parents to probands. Additionally, to detect possible bias or systematic issues in the analyses, we also assessed the over-transmission of polygenic risks generated from PGC3-SCZ^5^ and LDL from the ENGAGE Consortium^39^ as positive and negative controls, respectively. Previous studies suggest that there is no genetic correlation or causal relationship between LDL and major psychiatric disorders, making LDL an appropriate comparison phenotype in which no inflation of psychiatric PRS would be expected^46^. Details of the PRS construction for PGC3-SCZ and LDL in the ISHDSF sample are provided elsewhere^10^ The pTDT deviation scores were generated for each multiplex family by subtracting the mean parental polygenic risks from the proband polygenic risks. The pTDT deviation scores were then standardized by dividing them by the cohort-specific mean parental polygenic risks standard deviations. To test whether the mean pTDT deviation was significantly greater than zero, representing an over-transmission of polygenic risks from parents to probands, a one-sided, one-sample *t*-test was employed.

### Statistical analyses

Statistical analyses were carried out using a mixed-effects logistic regression model fitted by maximum likelihood using the GMMAT package in R^47^. To account for the high degree of relatedness among the subjects, genomic relationship matrix (GRM) was calculated using LDAK^48^ and included in the statistical models as a random effect. While none of the principal components (PCs) showed a significant association with genotype arrays or sites, in order to account for other possible batch and site effects, we also included genotyping platform and site as additional covariates, with more details provided in the Supplementary Materials.

Principal component analysis was consistent with all subjects in the sample having European ancestry (Supplementary Figs. 7–9), but to account for fine-scale structure within the Irish population^41^ (Supplementary Fig. 10) the top 10 ancestry PCs were also included as covariates in the analysis. The final mixed models included GRM as a random effect, with the top 10 PCs, genotyping platform, genotyping site, and sex as fixed effect covariates. All statistical analyses followed the hypothesis that members of multiplex SCZ families and sporadic cases used as a replication cohort have a significantly higher PRS compared to population controls and all PRS underwent Z-score normalization. The results were adjusted for multiple testing correction using the Holm method. While less stringent than Bonferroni, the family-wise error rate for the Holm method is similar to Bonferroni^49^, making it suitable for multiple testing correction in modestly sized cohorts such as our sample.

### Polygenic overlap analysis

We used MiXeR framework^16^ to quantify the polygenicity and the polygenic overlap of SCZ with BIP and MDD. MiXeR uses GWAS summary statistics to estimate the polygenicity of each phenotype and constructs a bivariate Gaussian mixture model to estimate the number of shared and unique variants that explains 90% of SNP heritability for each GWAS. We also estimated the heritability of the derived factors underlying BIP and MDD using LD Score Regression^42^ as shown in Supplementary Table 8.

## Supplementary information

Supplementary Materials

## Data Availability

The scripts used in this study are available on GitHub: https://github.com/ahangarim/SCZ_Fam_analysis. We made use of various freely available software tools in this study: Plink2: https://www.cog-genomics.org/plink/2.0/ GenomicSEM: https://github.com/GenomicSEM/GenomicSEM PRS-CS: https://github.com/getian107/PRScs LDSC: https://github.com/bulik/ldsc GMMAT: https://github.com/hanchenphd/GMMAT LDAK: http://dougspeed.com/ldak/ MiXeR: https://github.com/precimed/mixer pTDT: https://github.com/ypaialex/ptdt GWAS summary statistics for schizophrenia, bipolar disorder, major depression, and LDL are publicly available:PGC3-SCZ: https://www.med.unc.edu/pgc/download-results/ PGC3-BIP: https://www.med.unc.edu/pgc/download-results/ PGC2-MDD-UKB Meta-analysis: https://datashare.ed.ac.uk/handle/10283/3203 LDL: http://diagram-consortium.org/2015_ENGAGE_1KG/ Leave-N-Out schizophrenia summary statistics excluding the Irish individuals and GWAS-by-subtraction summary statistics generated in this study can be provided upon request by contacting the corresponding author.

## References

[1] Kendler KS (1985). Diagnostic approaches to schizotypal personality disorder: a historical perspective. Schizophr. Bull..

[2] Kety SS, Rosenthal D, Wender PH, Schulsinger F, Jacobsen B (1975). Mental illness in the biological and adoptive families of adopted individuals who have become schizophrenic: a preliminary report based on psychiatric interviews. Proc. Annu. Meet. Am. Psychopathol. Assoc..

[3] Wray NR (2018). Genome-wide association analyses identify 44 risk variants and refine the genetic architecture of major depression. Nat. Genet..

[4] Mullins N (2021). Genome-wide association study of more than 40,000 bipolar disorder cases provides new insights into the underlying biology. Nat. Genet..

[5] Trubetskoy V (2022). Mapping genomic loci implicates genes and synaptic biology in schizophrenia. Nature.

[6] Lee PH, Feng YCA, Smoller JW (2021). Pleiotropy and cross-disorder genetics among psychiatric disorders. Biol. Psychiatry.

[7] Kendler KS (1996). Irish study of high-density schizophrenia families: field methods and power to detect linkage. Am. J. Med. Genet..

[8] Asarnow RF (2001). Schizophrenia and schizophrenia-spectrum personality disorders in the first-degree relatives of children with schizophrenia: the UCLA Family study. Arch. Gen. Psychiatry.

[9] Kendler KS, Neale MC, Walsh D (1995). Evaluating the spectrum concept of schizophrenia in the Roscommon Family Study. Am. J. Psychiatry.

[10] Ahangari, M. et al. Evaluating the role of common risk variation in the recurrence risk of schizophrenia in multiplex schizophrenia families. Transl. Psychiatry 12, 291, https://www.nature.com/articles/s41398-022-02060-3 (2022). 10.1038/s41398-022-02060-3 PMC9304393 35864105

[11] Bigdeli TB (2014). Molecular validation of the schizophrenia spectrum. Schizophr. Bull..

[12] Demange PA (2021). Investigating the genetic architecture of noncognitive skills using GWAS-by-subtraction. Nat. Genet..

[13] Grotzinger AD (2019). Genomic structural equation modelling provides insights into the multivariate genetic architecture of complex traits. Nat. Hum. Behav..

[14] Weiner D (2017). Polygenic transmission disequilibrium confirms that common and rare variation act additively to create risk for autism spectrum disorders. Nat. Genet.

[15] Kendler KS (1993). The Roscommon family study: II. The risk of nonschizophrenic nonaffective psychoses in relatives. Arch. Gen. Psychiatry..

[16] Frei O (2019). Bivariate causal mixture model quantifies polygenic overlap between complex traits beyond genetic correlation. Nat. Commun..

[17] Howard DM (2019). Genome-wide meta-analysis of depression identifies 102 independent variants and highlights the importance of the prefrontal brain regions. Nat. Neurosci..

[18] Kendler KS, Gruenberg AM, Tsuang MT (1985). Psychiatric illness in first-degree relatives of schizophrenic and surgical control patients: a family study using DSM-III criteria. Arch. Gen. Psychiatry.

[19] Jablensky A (2001). Classification of nonschizophrenic psychotic disorders: a historical perspective. Curr. Psychiatry Rep..

[20] Kendler KS (1993). The Roscommon family study: IV. Affective illness, anxiety disorders, and alcoholism in relatives. Arch. Gen. Psychiatry..

[21] Smeland OB, Frei O, Dale AM, Andreassen OA (2020). The polygenic architecture of schizophrenia — rethinking pathogenesis and nosology. Nat. Rev. Neurol..

[22] Ahangari M (2022). Genome-wide analysis of schizophrenia and multiple sclerosis identifies shared genomic loci with mixed direction of effects. Brain Behav. Immun..

[23] Cheng W (2021). Genetic association between schizophrenia and cortical brain surface area and thickness. JAMA Psychiatry.

[24] Holland D (2020). Beyond SNP heritability: polygenicity and discoverability of phenotypes estimated with a univariate Gaussian mixture model. PLoS Genet.

[25] Spielman RS, McGinnis RE, Ewens WJ (1993). Transmission test for linkage disequilibrium: the insulin gene region and insulin-dependent diabetes mellitus (IDDM). Am. J. Hum. Genet..

[26] Rees E (2020). De novo mutations identified by exome sequencing implicate rare missense variants in SLC6A1 in schizophrenia. Nat. Neurosci..

[27] Andlauer TFM (2021). Bipolar multiplex families have an increased burden of common risk variants for psychiatric disorders. Mol. Psychiatry..

[28] Szatkiewicz J (2019). The genomics of major psychiatric disorders in a large pedigree from Northern Sweden. Transl. Psychiatry.

[29] de Jong S (2018). Applying polygenic risk scoring for psychiatric disorders to a large family with bipolar disorder and major depressive disorder. Commun. Biol.

[30] Halvorsen M (2021). Elevated common variant genetic risk for tourette syndrome in a densely-affected pedigree. Mol Psychiatry..

[31] Davis LK (2013). Partitioning the heritability of tourette syndrome and obsessive compulsive disorder reveals differences in genetic architecture. PLoS Genet.

[32] Yang Z (2021). Investigating shared genetic basis across tourette syndrome and comorbid neurodevelopmental disorders along the impulsivity-compulsivity spectrum. Biol. Psychiatry..

[33] Arnold PD (2018). Revealing the complex genetic architecture of obsessive-compulsive disorder using meta-analysis. Mol. Psychiatry..

[34] Demontis D (2019). Discovery of the first genome-wide significant risk loci for attention deficit/hyperactivity disorder. Nat. Genet..

[35] Cheng S (2022). Exome-wide screening identifies novel rare risk variants for major depression disorder. Mol. Psychiatry..

[36] Palmer DS (2022). Exome sequencing in bipolar disorder identifies AKAP11 as a risk gene shared with schizophrenia. Nat. Genet..

[37] Marshall CR (2017). Contribution of copy number variants to schizophrenia from a genome-wide study of 41,321 subjects. Nat. Genet..

[38] Donnelly P (2012). Genome-wide association study implicates HLA-C*01:02 as a risk factor at the major histocompatibility complex locus in schizophrenia. Biol. Psychiatry..

[39] Surakka I (2015). The impact of low-frequency and rare variants on lipid levels. Nat. Genet..

[40] Loh PR (2016). Reference-based phasing using the Haplotype Reference Consortium panel. Nat. Genet..

[41] Gilbert E (2017). The Irish DNA Atlas: revealing fine-scale population structure and history within Ireland. Sci. Rep.

[42] Bulik-Sullivan, B. K. & Neale, B. M. LD score regression distinguishes confounding from polygenicity in GWAS. Nat. Genet. 47, 291–295, http://www.standard.co.uk/news/the-parking-meter-clocks-up-50-years-6920973.html (2015). 10.1038/ng.3211 PMC4495769 25642630

[43] Ge T, Chen CY, Ni Y, Feng YCA, Smoller JW (2019). Polygenic prediction via Bayesian regression and continuous shrinkage priors. Nat. Commun.

[44] Clarke L (2017). The international Genome sample resource (IGSR): a worldwide collection of genome variation incorporating the 1000 Genomes Project data. Nucleic Acids Res..

[45] Ni G (2021). A comparison of ten polygenic score methods for psychiatric disorders applied across multiple Cohorts. Biol. Psychiatry..

[46] Bulik-Sullivan B (2015). An atlas of genetic correlations across human diseases and traits. Nat. Genet..

[47] Chen H (2016). Control for population structure and relatedness for binary traits in genetic association studies via logistic mixed models. Am. J. Hum. Genet..

[48] Speed D, Hemani G, Johnson MR, Balding DJ (2012). Improved heritability estimation from genome-wide SNPs. Am. J. Hum. Genet..

[49] Aickin M, Gensler H (1996). Adjusting for multiple testing when reporting research results: the Bonferroni vs Holm methods. Am. J. Public Health..

