# Supplementary material for: Examining the source of increased bipolar disorder and major depressive disorder common risk variation burden in multiplex schizophrenia families
Source: Schizophrenia (Heidelb). 2022 Nov 25;8(1):106. doi: 10.1038/s41537-022-00317-w (PMC9700852; doi:10.1038/s41537-022-00317-w)
Supplement: Supplementary file 1 — Supplementary Materials [file 41537_2022_317_MOESM1_ESM.docx]

**Supplementary materials for:**

**Examining the source of increased bipolar disorder and major depressive disorder common risk variation burden in multiplex schizophrenia families**

Ahangari et al (2022)

Supplementary notes on pages 2-7

Supplementary tables on pages 8-11

Supplementary figures on pages 12-21

References on page 22

**Imputation Quality Control**

We used the following Michigan Imputation Server (1) to impute the genotypes and perform the initial quality control (QC) on them:

First, chunks of 20 Mb were created from the uploaded genotypes. Then, on each chunk, the followings were checked:

1. Number of valid variants in the chunk. A variant was considered to be valid when it is included in the reference panel.
2. Number of variants found in the reference panel for the chunk, where at least 50% of the variants must be included in the reference panel.
3. Sample call rate for the chunk, where at least 50% of the variants must be called for each sample.

Chunks were excluded if number of variants < 3, overlap is < 50% and sample call rate is < 50%.

Second, the followings were checked on the variant level:

1. Only A, C, G, T alleles are allowed
2. Alternate allele frequency (AF) is calculated and all markers with AF > 0.5 are flagged.
3. SNP call rate is calculated
4. Chi square for each variant is calculated by using reference panel vs study data.
5. Allele switches are determined by comparing reference and alternate allele of the reference panel vs study data with A/T and C/G variants ignored.
6. After removing possible allele switches, strand flips are determined by flipping and comparing reference and alternate alleles from the reference panel vs study data.
7. Determine both allele switches and strand flips by combining steps 5 and 6.

Variants are excluded if alleles other than A, C, G, T are observed, duplicates are observed, indels, monomorphic sites, allele mismatch between reference panel vs study data and SNP call rate of < 90%.

Third, the followings are checked at sample level:

1. For chromosomes 1 to 22 analyzed here, a chunk is excluded if one sample has a call rate < 50 % with only the complete chunks excluded and not samples.
2. Liftover is performed if the build of the input data and reference panel does not match.

Following the above steps, we applied the r2 threshold of 0.3 which removed >70% of poorly imputed SNPs at the cost of < 0.5% well imputed SNPs and minor allele frequency of 1%.

Supplementary figures 1-3 show the allele frequency correlation of arrays versus HRC reference panel.

**Imputation quality across the arrays**

After performing the QC steps described in the main manuscript under “Imputation” section in the methods, and supplemental information provided above about imputation procedure using the Michigan Imputation Server, 9,298,012 SNPs in the Illumina Array, 11,080,279 SNPs in the Affymetrix Array, and 11,081,999 SNPs in the PsychArray remained for analysis. In total, 9,008,825 of these SNPs were shared across all three arrays. We merged these shared SNPs with HapMap3 SNPs as described in the main manuscript under “Polygenic risk score construction” section in the methods which left us with 943,020 high quality SNPs with r^2^ of at least 0.96 across all 3 arrays for PRS construction, as shown in supplementary table 2 below.

**Principal component analysis**

The principal component analysis (PCA) was performed using PLINK (2). We used the 1000 Genomes phase 3 data set populations as the background for our continental PCA analysis (3). Variants not on the coding strand from the 1000 Genomes Phase 3 dataset were first removed and only the variants with the minor allele frequency of more than 0.10 were retained. Next, all the other variants for each chromosome were pruned using the flag --indep 50 5 1.5. This left us of 349,512 variants on the 1000 Genomes Phase 3. High quality Imputed genotypes with r^2^ value of more than 0.99 were extracted from each of the three arrays and merged with the 349,512 variants from the 1000 Genomes Phase 3 dataset to get a list of all the available SNPs across all platforms and 1000 Genomes Phase 3 for PCA analysis. The total number of variants shared across all 3 platforms and the 1000 Genomes Phase 3 was 164,307 variants. We then used –pca flag in PLINK V.1.9 to calculate the top 20 PCAs.

**Covariates and accounting for possible batch effects**

Subjects used in this study were genotyped on 3 different arrays as shown in the supplementary table 1. These 3 arrays are the Affymetrix V.6.0. Array, Illumina 610-Quad Array, and the Illumina Infinium PsychArray V.1.13. Sporadic cases and population controls on the Affymetrix V 6.0 array were genotyped at either the Broad Institute or Affymetrix. The ISHDSF sample were genotyped on the Illumina 610-Quad array by the Illumina. An additional set of case-controls and the ISHDSF subjects were genotyped on the PsychArray at Mount Sinai. A number of individuals for whom we already had genotypes available on the Illumina array or the Affymetrix array, were genotyped again on the PsychArray for quality control purposes. We compared and contrasted the polygenic risks constructed for these duplicate individuals and observed no significant differences among them, with high correlation (all > 0.94) with no significant difference in imputation quality across the arrays (Supplementary Figure 8). Additionally, the principal components (PCs) showed no significant association with any of the genotyping batches or sites, indicating that there is no systematic differences or biases between the three arrays. However, to account for other possible batch or site effects, we included each of the platforms and genotyping sites as covariates in all models. In addition, although PCA shows that all the individuals analyzed in this study are of European ancestry, due to fine-scale population structure in the population of the island of Ireland, we included the top 10 PCs as covariates in our analyses.

**GWAS-by-subtraction**

GWAS-by-subtraction method specifies models within the genomicSEM framework.

Graphical representation of the models are provided in supplementary figure 4 where circles represent latent components of the model (*SCZ factors* and *Affective factors*) and squares represent the observed SNPs and genetic components of the original GWAS phenotypes (leave-N-out SCZ, BIP or MDD). Similar to structural equation modeling conventions, single headed arrows represent linear regression associations pointing from the independent variable to the dependent variable and two-headed arrows represent variances and covariance relationships. Full set of algebraic equations for the GWAS-by-subtraction model are provided elsewhere by Demange et al (4). GWAS-by-subtraction model assumes that all genetic effects on SCZ are also impacting BIP (or MDD) to some degree. This assumption should be reasonable, given that SCZ and BIP (or MDD) have strong genetic correlations with each other. Therefore, for any pair of traits used in a GWAS-by-subtraction analysis, a similar consideration needs to be made (like the case with BIP or MDD here).

In brief, the regression equations composing GWAS-by-subtraction model in this study are:

**Bipolar disorder:**

SCZ = λ_1_SCZ factor BIP = λ_2_ SCZ factor + λ_3_Affectiv*e* factor

SCZ factor = *𝛃*_1_SNP + 𝜇_SCZ factor_

Affective factor= *𝛃*_2_SNP + 𝜇_Affective factor_

**Major depressive disorder:**

SCZ = λ_1_SCZ factor MDD = λ_2_SCZ factor + λ_3_Affective factor

SCZ factor = *𝛃*_1_SNP + 𝜇_SCZ factor_

Affective factor= *𝛃*_2_SNP + 𝜇_Affective factor_

SNP filtering for GWAS-by-subtraction

Similar to other genomicSEM models, or software like MTAG, GWAS-by-subtraction framework assumes that genetic covariance estimated from LDSC can be generalized to each of the SNPs individually. When running GWAS-by-subtraction, SNPs with MAF < 0.01 according to the SNPs from the HapMap3 reference panel are excluded and SNPs are also filtered based on their presence in the 1000 Genomes phase one. Since *SCZ* and *Affective factors* GWAS results are obtained from the GWAS-by-subtraction analysis of SCZ with either BIP or MDD GWAS, only SNPs that are present in both SCZ and BIP or MDD are present in the *SCZ* and *Affective factor* GWAS generated from GWAS-by-subtraction. This left us with 6,361,243 SNPs for BIP models and 6,599,052 SNPs for MDD models.

Effective sample size calculation

We used a method suggested by Mallard et al (5) to calculate the effective sample size calculation

First, we restrict to SNPs MAF of 40% and 10% using the following script in R:

*df <- subset(df, df$MAF <= 0.4 & df$MAF >= 0.1)*

Where df is the GWAS-by-subtraction file.

Since we have performed a Cholesky model in the GWAS-by-subtraction models, we need to also adjust the estimates ‘*est’* by multiplying them by the residual heritability for each GWAS-by-subtraction model as shown in the description for supplementary figure 4 and supplementary table 3 ( λSCZfactor-BIP, λAffective-BIP, λSCZfactor-MDD and λAffective-MDD)

We then calculate the effective sample size using the following script in R:

*effective_n <- (mean((df$Z_Estimate/df$est*λ)^2/(2*df$MAF*(1-df$MAF))))*

Where Z_Estimate is the Z statistic from the GWAS and est is the path estimates and λ is the residual for each GWAS.

This formula is prone to error for SNPs with low MAF. Therefore, it is suggested by Mallard et al that we set a lower and upper MAF limit of approximately 10% and 40% when estimating the effective sample size.

**Supplementary Tables:**

**Supplementary Table 1:** Full bipolar disorder PRS results for ISHDSF diagnostic categories versus population controls. *p*-values were adjusted using holm method in R.

**Supplementary Table 2:** Full major depressive disorder PRS results for ISHDSF diagnostic categories versus population controls. *p*-values were adjusted using holm method in R.

**Supplementary Table 3:** GWAS-by-subtraction PRS results for each underlying factor. *p*-values were adjusted using holm method in R.

**Supplementary Table 4:** Concentric comparison of GWAS-by-subtraction and univariate bipolar and major depressive disorder PRS in the ISHDSF sample versus population controls. *p*-values were adjusted using holm method in R.

| **Narrow + Intermediate** | |  |
| --- | --- | --- |
| PRS | PVAL | Adj_PVAL |
| BIP | 2.44E-24 | 2.68E-23 |
| SCZ factor in BIP | 1.51E-59 | 2.27E-38 |
| Affective factor in BIP | 0.749 | 1 |
| MDD | 6.43E-08 | 4.50E-07 |
| SCZ factor in MDD | 1.43E-55 | 1.86E-34 |
| Affective factor in MDD | 0.0931 | 0.5586 |
| **Narrow + Intermediate + Broad** | |  |
| PRS | PVAL | Adj_PVAL |
| BIP | 1.77E-25 | 2.12E-24 |
| SCZ factor in BIP | 1.44E-67 | 2.30E-46 |
| Affective factor in BIP | 0.475 | 1 |
| MDD | 3.01E-08 | 2.41E-07 |
| SCZ factor in MDD | 1.21E-58 | 1.69E-41 |
| Affective factor in MDD | 0.219 | 0.876 |
| **Narrow + Intermediate + Broad + Very Broad** | | |
| PRS | PVAL | Adj_PVAL |
| BIP | 3.19E-24 | 3.19E-23 |
| SCZ factor in BIP | 4.83E-73 | 8.69E-52 |
| Affective factor in BIP | 0.572 | 1 |
| MDD | 4.60E-09 | 4.14E-08 |
| SCZ factor in MDD | 8.69E-69 | 1.48E-47 |
| Affective factor in MDD | 0.148 | 0.74 |

**Supplementary Table 5:** Description of the arrays used in this study. Number of individuals and pre/post imputation SNPs on each array are provided.

| **Array** | **ISGC** | **ISHDSF** | **N SNP’s QC’d pre-imputation** | **N imputed SNPs QC’d** |
| --- | --- | --- | --- | --- |
| **Illumina 610-Quad**   - **Narrow** - **Intermediate** - **Broad** - **Very Broad** - **Unaffected** | **NA** | **830**   - **430** - **102** - **50** - **36** - **211** | **557,373** | **9,298,012** |
| **Affymetrix V.60**   - **Case** - **Control** | **1,730**   - **1,509** - **1,731** | **NA** | **686,646** | **11,080,279** |
| **Infinium psychArray v.1.13**   - **Narrow** - **Intermediate** - **Broad** - **Very Broad** - **Unaffected** - **Case** - **Control** | **1,296**   - **716** - **580** | **176**   - **39** - **10** - **1** - **105** - **21** | **384,389** | **11,081,999** |

**Supplementary Table 6:** HapMap3 SNPs across the 3 arrays used for PRS construction. Mean imputation quality scores (SD) are provided on the 4^th^ column for the imputed SNPs.

| **Array** | **Genotyped** | **Imputed** | **Mean r^2^ (SD)** |
| --- | --- | --- | --- |
| **Affymetrix V.60** | 443,872 | 499,148 | 0.98 (0.041) |
| **Illumina 610-Quad** | 414,052 | 528,968 | 0.98 (0.035) |
| **Infinium PsychArray** | 338,265 | 604,755 | 0.96 (0.056) |

**Supplementary Table 7:** Model parameters for GWAS-by-subtraction analyses of BIP and MDD.

**Supplementary Table 8:** Heritability estimates and LD score intercepts for GWAS-by-subtraction results. Note that heritability estimates are on observed scale.

**Supplementary Figures:**

**
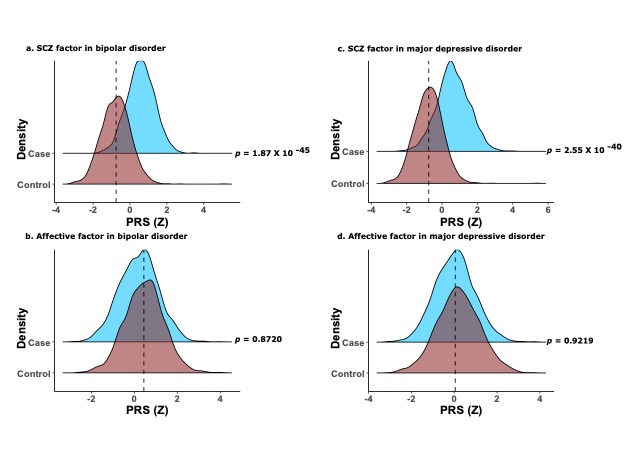
**

**Supplementary Figure 1:** Density plots showing the distribution of *SCZ* factor and *Affective* factor PRS results in the replication schizophrenia (SCZ) sample from the ISGC. Dotted line shows the mean PRS value for population controls. Panels **a** and **b** show the distribution of *SCZ* and *Affective* factor PRS for bipolar disorder (BIP). Panels **c** and **d** show the distribution of *SCZ* and *Affective* factor PRS for MDD. One-sided *p*-values after multiple testing correction are provided on the right side of each panel. All comparisons follow the hypothesis that sporadic SCZ cases have higher PRS compared to population controls.


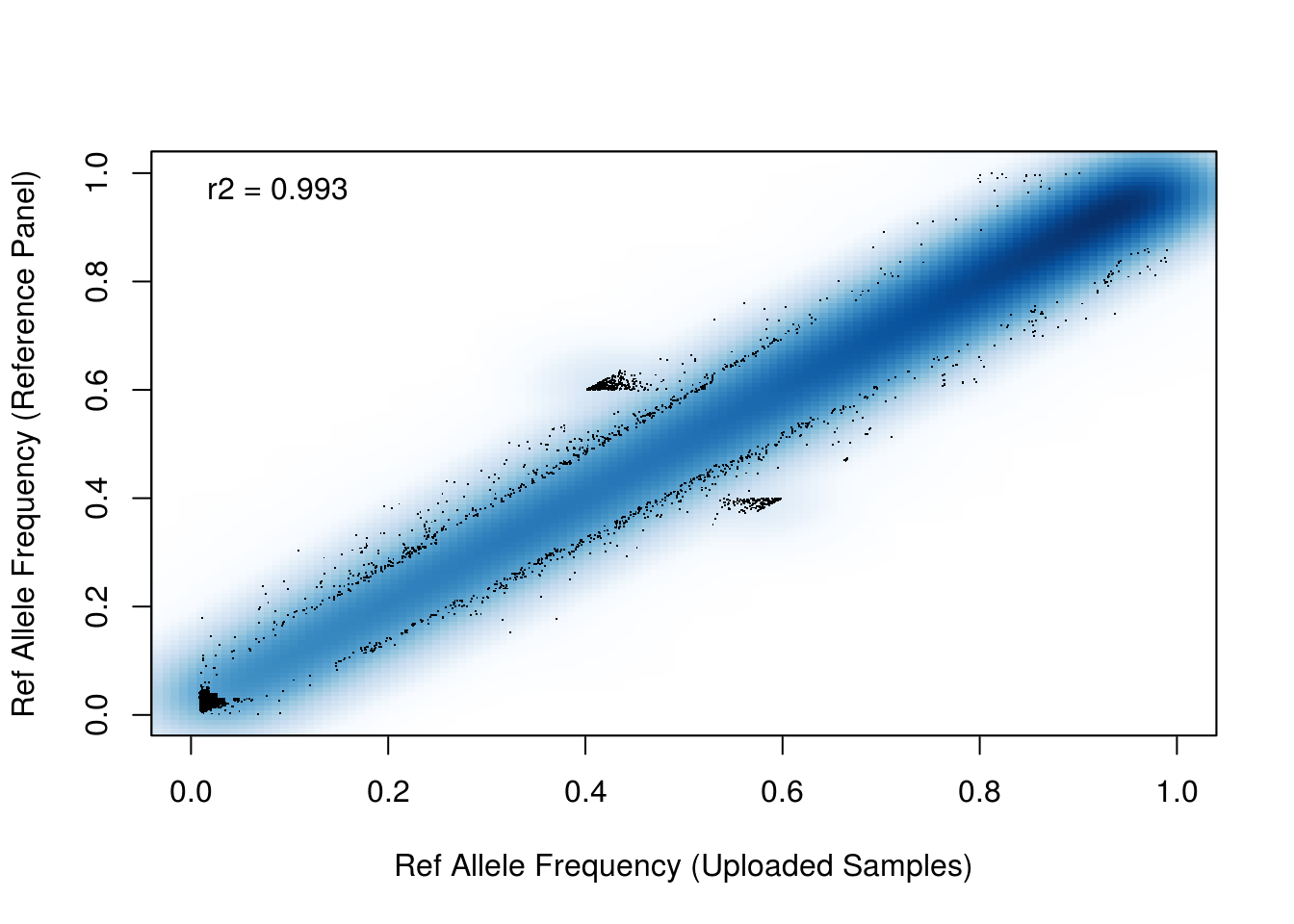


**Supplementary Figure 2:** Allele frequency correlation for the Affymetrix Array vs HRC reference panel. The plot shows the densities of frequencies falling into each part, with the first 5000 points from areas of lowest regional densities plotted.


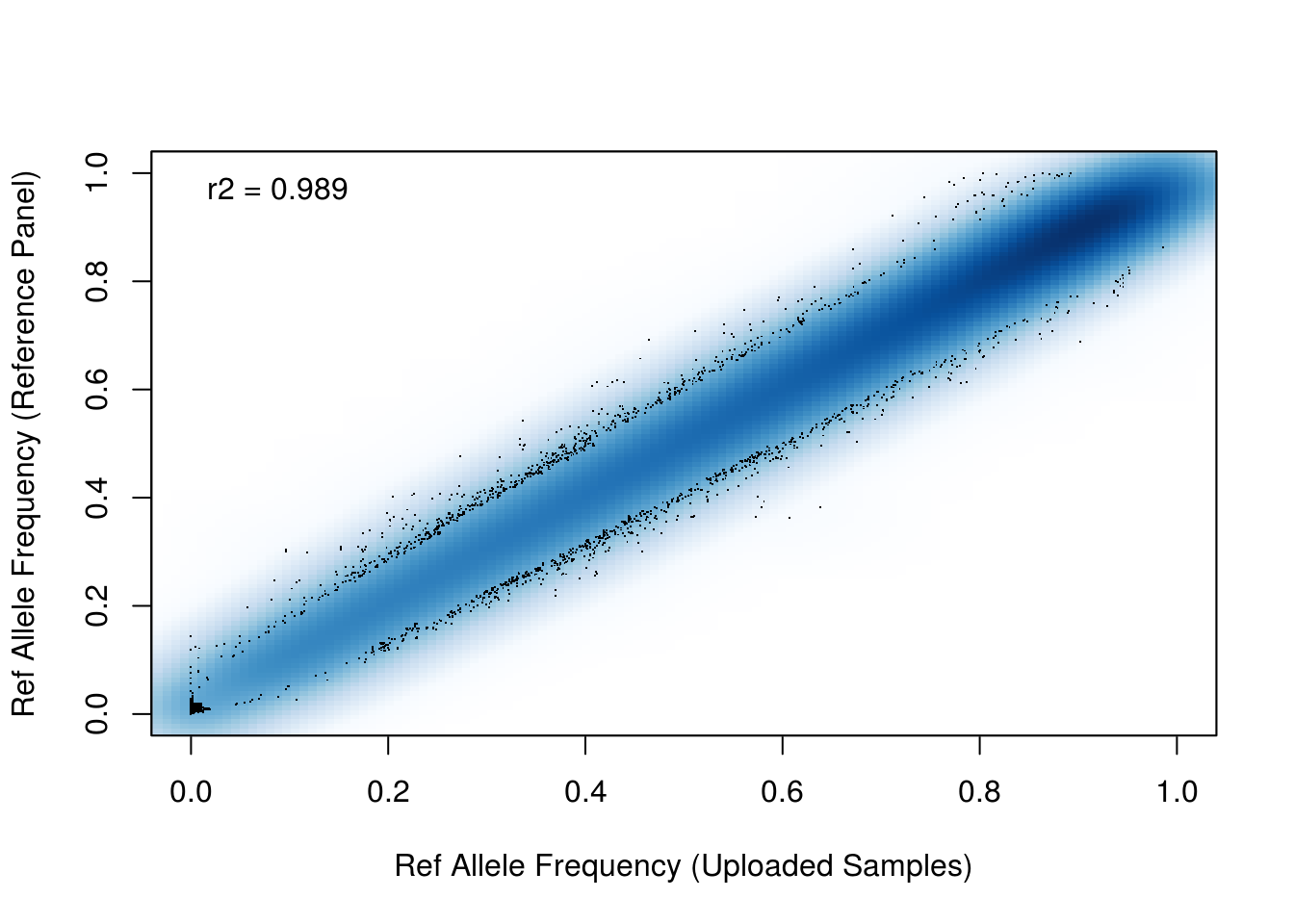


**Supplementary Figure 3:** Allele frequency correlation for the Illumina Array vs HRC reference panel. The plot shows the densities of frequencies falling into each part, with the first 5000 points from areas of lowest regional densities plotted.


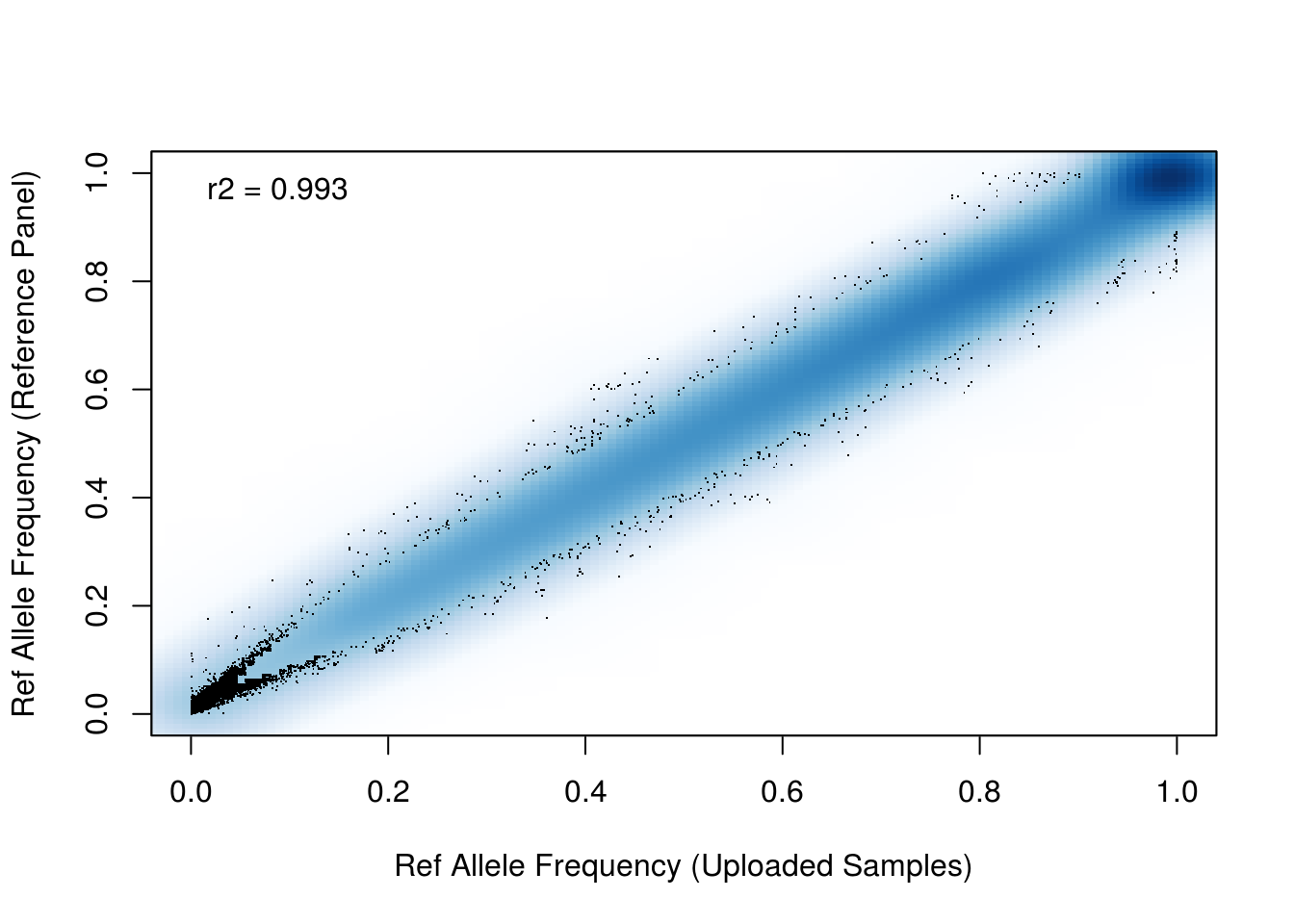


**Supplementary Figure 4:** Allele frequency correlation for the psychArray vs HRC reference panel. The plot shows the densities of frequencies falling into each part, with the first 5000 points from areas of lowest regional densities plotted.


**Supplementary Figure 5:** Comparison of imputation quality across the three arrays. Variants were binned according to their minor allele frequency and imputation R2 averaged across variants in each bin. All three arrays were imputed to the Haplotype Reference Consortium Reference Panel on the Michigan Imputation Server.


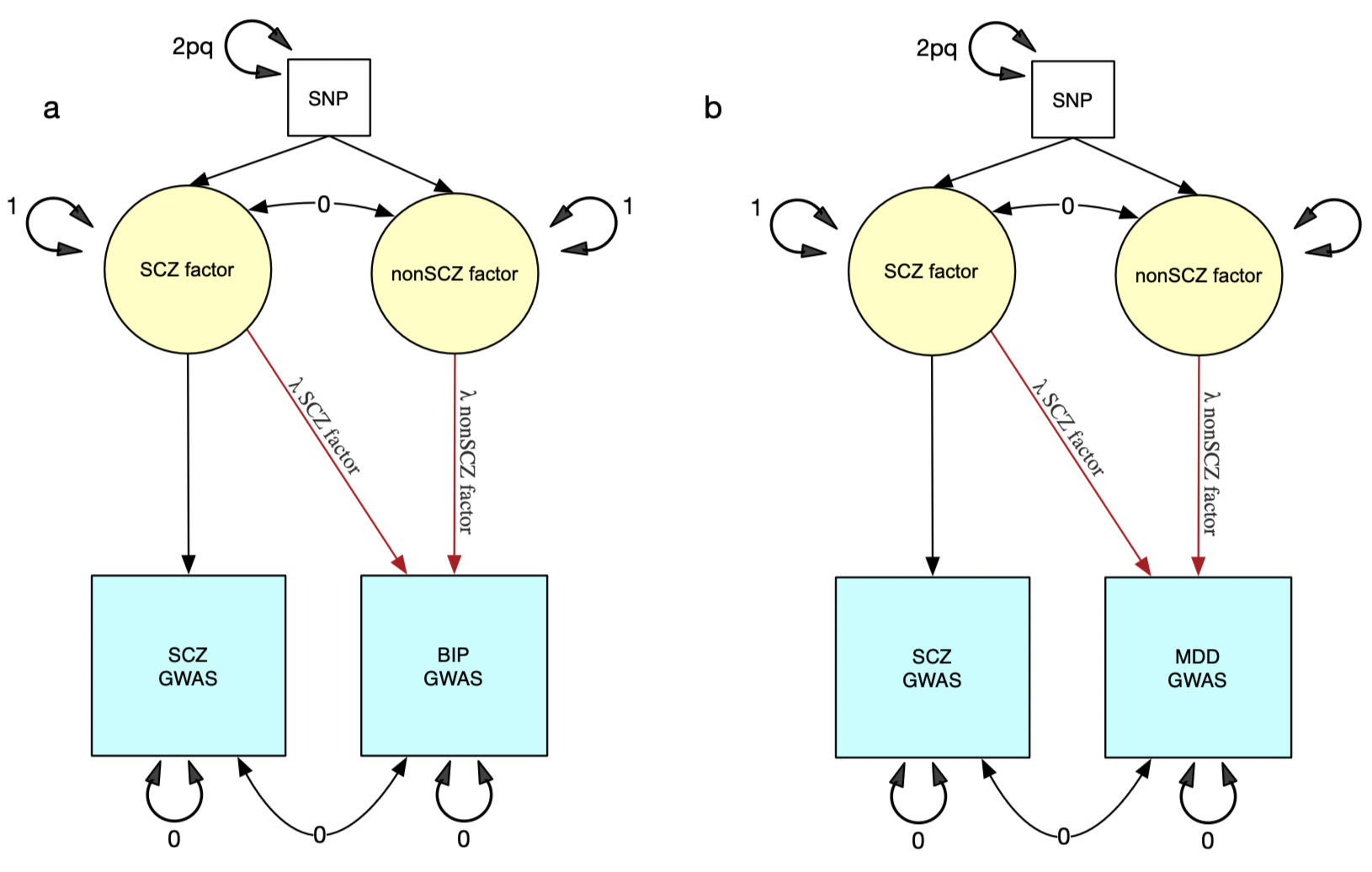


**Supplementary Figure 6:** The Cholesky model fitted within the GenomicSEM for GWAS-by subtraction models. Circles represent latent variables and squares represent observed variables. **a.** Path diagram for BIP GWAS-by-subtraction analysis. **b.** Path diagram for MDD GWAS-by-subtraction analysis. The genetic covariances between SCZ and BIP or MDD were measured based on their respective GWAS summary statistics using LDSC within genomicSEM in R. The covariance between SCZ GWAS and BIP or MDD GWAS are fixed at 0. The variance for SNPs is fixed at 2pq, with *p* being the reference allele frequency and *q* being the alternative allele frequency inferred from the 1000 Genomes Project. The residual variance for SCZ and BIP or MDD is fixed to be 0, and the variance in latent factors are set to 1. This was done in order to have all the variance explained by the two latent factors which we call *SCZ* factor and *affective* factor (labeled nonSCZ factor in the figure). The observed GWAS for SCZ and BIP or MDD (turquoise squares) were regressed on the two latent factors, with path estimates of λ_SCZfactor - BIP_= 0.2339; λ_nonSCZfactor - BIP_= 0.2502; and λ_SCZfactor - MDD_= 0.0934; λ_nonSCZfactor - MDD_= 0.2715. The two latent factors were then regressed on each SNP for GWAS-by-subtraction analysis to produce four new sets of summary statistics for each latent factor represented with a yellow circle. Note that since both the ISHDSF and ISPGC samples are part of the PGC3-SCZ mega-analysis, leave-N-out SCZ summary statistics excluding the Irish components were used for SCZ signals to ensure that there is no upwards bias in the results.

**Supplementary Figure 7:** Continental PCA plot for the samples genotyped on the Affymetrix projected on the 1000 Genomes Phase 3 data. PC1 on the X-axis and PC2 on the Y-axis. Each color represents one of the ancestries. Black color represents the Irish sample.

**Supplementary Figure 8:** Continental PCA plot for the samples genotyped on the Illumina array projected on the 1000 Genomes Phase 3 data. PC1 on the X-axis and PC2 on the Y-axis. Each color represents one of the ancestries. Black color represents the Irish sample.

**Supplementary Figure 9:** Continental PCA plot for the samples genotyped on the psychArray projected on the 1000 Genomes Phase 3 data. PC1 on the X-axis and PC2 on the Y-axis. Each color represents one of the ancestries. Black color represents the Irish sample.

**Supplementary Figure 10:** Fine-scale PCA analysis of the Irish population. The fine-scale population structure captured for PC1 on the X-axis and PC2 on the Y-axis closely resembles the populations of the British Isles. Each color represents one of the arrays. Green represents population controls genotyped on the Affymetrix array. Blue represents ISHDSF genotypes on the Illumina array. Black represents ISHDSF or population controls genotyped on the psychChip. The top 10 PCs were used as covariates in the subsequent analyses.

**References:**

1. Das S, Forer L, Schönherr S, Sidore C, Locke AE, Kwong A, et al. Next-generation genotype imputation service and methods. Nat Genet. 2016;48(10):1284–7.

2. Purcell S, Neale B, Todd-Brown K, Thomas L, Ferreira MAR, Bender D, et al. PLINK: A Tool Set for Whole-Genome Association and Population-Based Linkage Analyses. Am J Hum Genet. 2007;81(3):559–75.

3. Auton A, Abecasis GR, Altshuler DM, Durbin RM, Bentley DR, Chakravarti A, et al. A global reference for human genetic variation. Nature. 2015;526(7571):68–74.

4. Demange PA, Malanchini M, Mallard TT, Biroli P, Cox SR, Grotzinger AD, et al. Investigating the genetic architecture of noncognitive skills using GWAS-by-subtraction. Nat Genet. 2021;53(1):35–44.

5. Mallard T, Linnér R, Grotzinger A, Sanchez-Roige S, Seidlitz J, Okbay A, et al. Multivariate GWAS of psychiatric disorders and their cardinal symptoms reveal two dimensions of cross-cutting genetic liabilities. bioRxiv. 2019;603134.
